# A Dictionary of Medical Science

**Published:** 1858-01

**Authors:** 


ART. VI.—*A Dictionary of Medical Science; containing a concise explanation of the various subjects and terms of Anatomy, Physiology, Pathology, Hygiene, Therapeutics, Pharmacology, Pharmacy, Surgery, Obstetrics, Medical Jurisprudence, etc. etc., with French and other Synonyms.* By ROBLEY DUNGLISON, M.D., LL.D., Prof. of Institutes of Medicine in the Jefferson Medical College. Fifteenth edition; revised and greatly enlarged. 8vo, pp. 992. Blanchard & Lea, Philadelphia, 1857.

PROFESSOR DUNGLISON'S Medical Dictionary has reached its *Fifteenth Edition!* What more need be said in its praise? The work is a monument of patient research, skilful judgment, and vast physical labor, that will perpetuate the name of the author more effectually than any possible device of stone or metal. Dr. Dunglison deserves the thanks not only of the American Profession, but of the whole medical world. The present edition, we are told in the Preface, contains an addition of *six thousand subjects and terms;* how and where they have been obtained are questions hardly less puzzling to those of us whose business it is to keep up with the current of medical literature, than to him whose reading is confined to the text-books of the schools.

